# Supplementary material for: Activation of a transient progenitor state in the epicardium is required for zebrafish heart regeneration
Source: Nat Commun. 2022 Dec 13;13:7704. doi: 10.1038/s41467-022-35433-9 (PMC9747719; doi:10.1038/s41467-022-35433-9)
Supplement: Supplementary file 1 — Supplementary Information [file 41467_2022_35433_MOESM1_ESM.pdf]

SUPPLEMENTARY INFORMATION

Activation of a transient progenitor state in the epicardium is required for zebrafish heart regeneration

Supplementary Figures

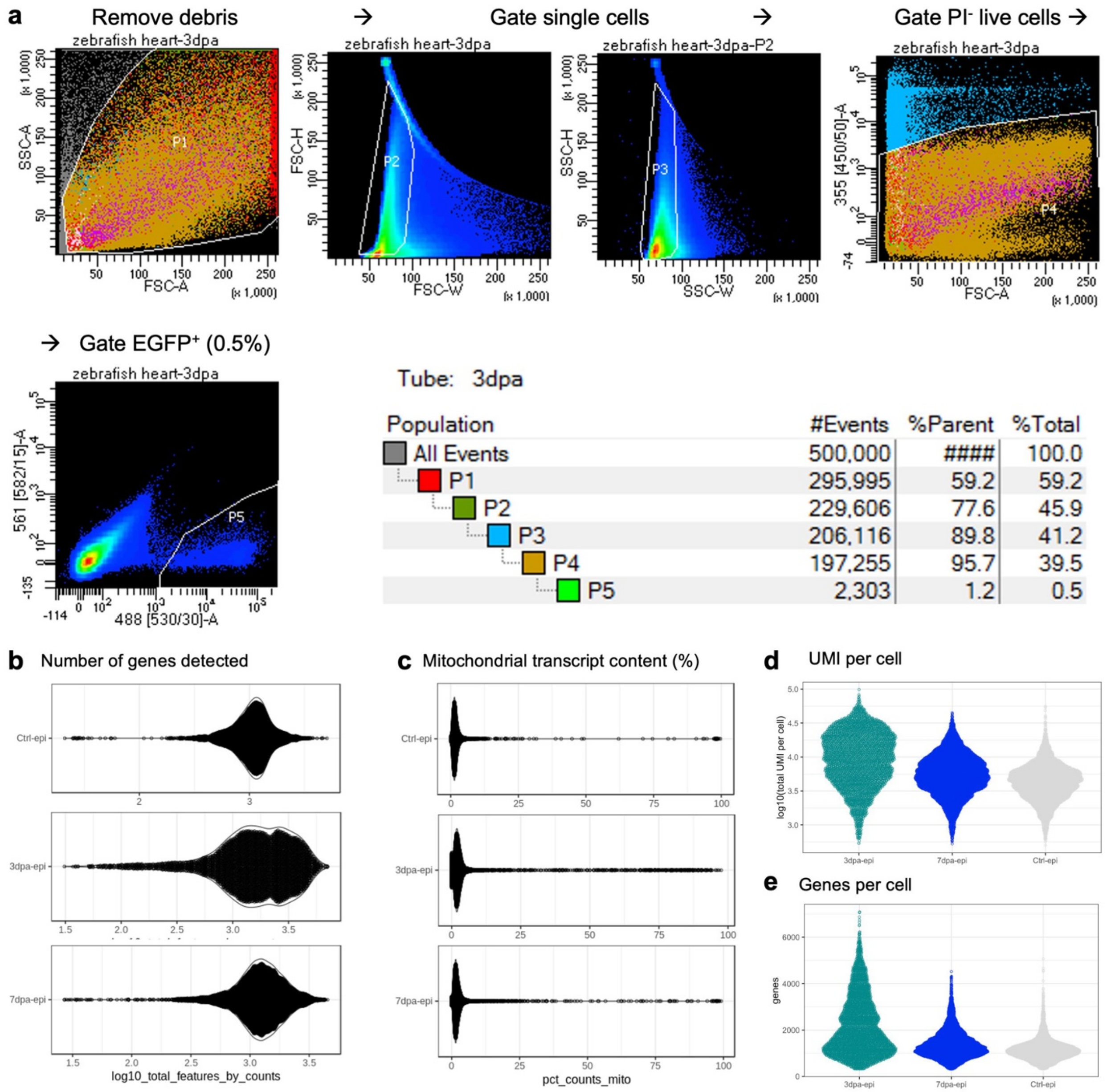

Supplementary Figure 1. FACS isolation of single *tcf21*$^{+}$ cells and QC of scRNAseq.
a Flow Cytometry plots showing the cell sorting process. About 0.5% of live (PI^{-}) GFP^{+}

cells were isolated from the ventricular dissociation. **b** The numbers of genes detected in each scRNA-seq sample. Each dot represents a single cell; the x-axis depicts the number of genes that were detected within a given cell. Most cells have evidence of at least 1,000 genes. **c** Mitochondrial transcript content (% transcripts originating from mitochondrially encoded genes). **d, e** Distribution of the UMI content (d) and genes (e) of each sample after removal of low-quality droplets and rarely captured genes. The 3 dpa sample yielded greater numbers of detected genes and, correspondingly, greater numbers of individual transcripts.

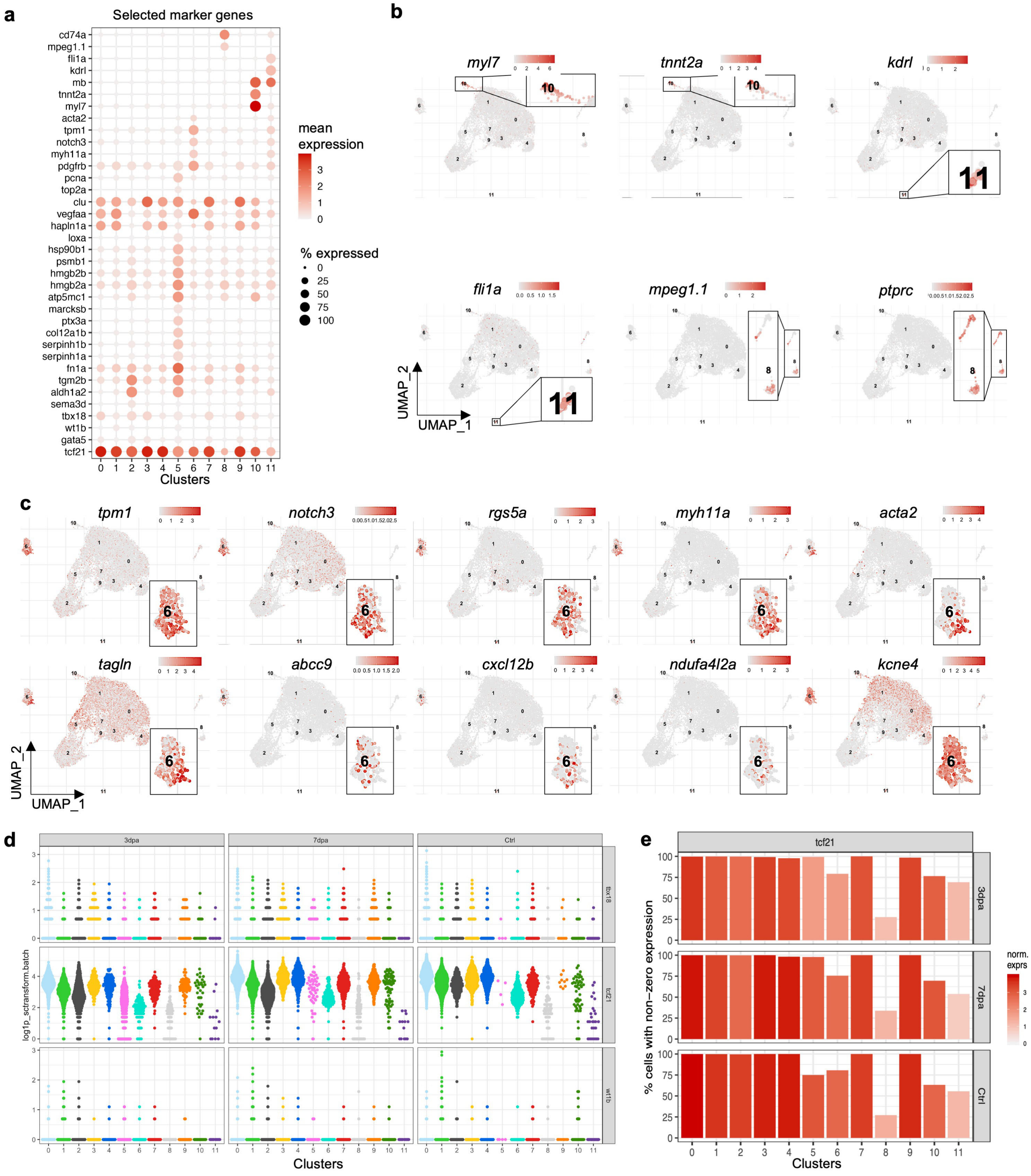

Supplementary Figure 2. Selected cluster markers. a Expression of selected cluster marker genes across different clusters. This dot plot depicts the abundance and expression magnitude of individual genes across cells of given clusters. The dot size represents the fraction of cells with at least one UMI of the specified gene. **b** Expression patterns of the selected non-epicardial genes within the UMAP projection (3 samples

combined). Magnified views are shown in frames. **c** Expression patterns of top mural cell markers on UMAPs (3 samples combined). Magnified views of cluster 6 are shown in frames. **d** A jitter plot showing relative expression levels (log1p_sctransform) of *tbx18*, *tcf21*, and *wt1b* in each cell across clusters (0-11) and conditions (Ctrl, 3 dpa, and 7 dpa). **e** Visualization of the magnitude and abundance of *tcf21* expression. Each bar's height represents the percentage of cells for which at least one fragment representing *tcf21* was detected. The color corresponds to the mean normalized expression value with dark red, indicating that numerous transcripts of *tcf21* were detected in the cells of a respective cluster (x-axis).

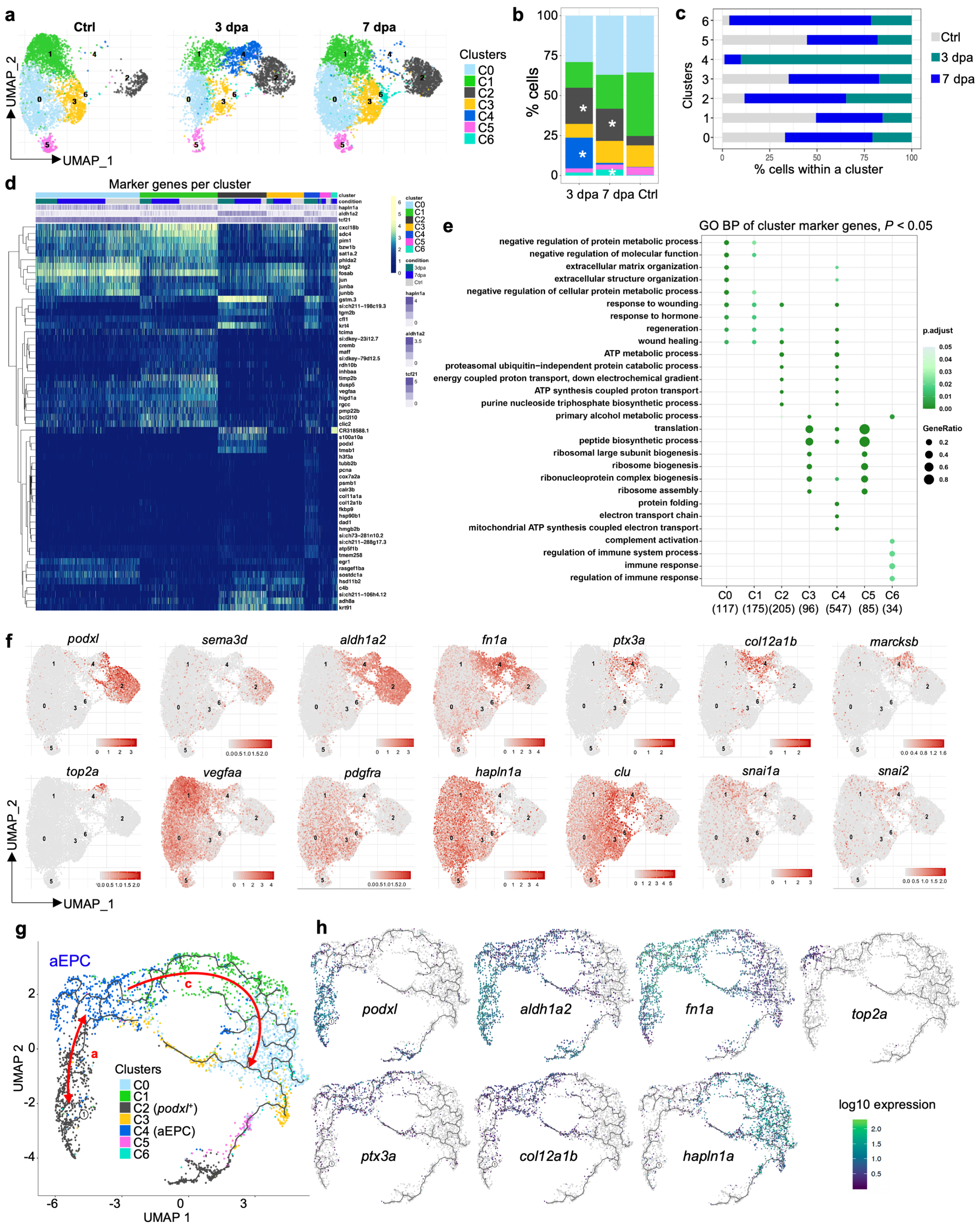

a
Ctrl
3 dpa
7 dpa
UMAP_2
UMAP_1
Clusters
C0
C1
C2
C3
C4
C5
C6
b
% cells
3 dpa 7 dpa Ctrl
c
Clusters
% cells within a cluster
Ctrl
3 dpa
7 dpa
d
Marker genes per cluster
e
GO BP of cluster marker genes, P < 0.05
negative regulation of protein metabolic process
negative regulation of molecular function
extracellular matrix organization
extracellular structure organization
negative regulation of cellular protein metabolic process
response to wounding
response to hormone
regeneration
wound healing
ATP metabolic process
proteasomal ubiquitin−independent protein catabolic process
energy coupled proton transport, down electrochemical gradient
ATP synthesis coupled proton transport
purine nucleoside triphosphate biosynthetic process
primary alcohol metabolic process
translation
peptide biosynthetic process
ribosomal large subunit biogenesis
ribosome biogenesis
ribonucleoprotein complex biogenesis
ribosome assembly
protein folding
electron transport chain
mitochondrial ATP synthesis coupled electron transport
complement activation
regulation of immune system process
immune response
regulation of immune response
p.adjust
GeneRatio
C0 (117)
C1 (175)
C2 (205)
C3 (96)
C4 (547)
C5 (85)
C6 (34)
f
podxl
sema3d
aldh1a2
fn1a
ptx3a
col12a1b
marcksb
top2a
vegfaa
pdgfra
hapln1a
clu
snai1a
snai2
g
aEPC
Clusters
C2 (podxl+)
C4 (aEPC)
UMAP 2
UMAP 1
h
podxl
aldh1a2
fn1a
top2a
ptx3a
col12a1b
hapln1a
log10 expression

Supplementary Figure 3. Subsets of the core epicardial clusters. a UMAP of cell clusters of the core population across samples after re-clustering following the removal of mural cells and presumed contaminants. Cells are re-clustered as Core 1 (or C1) to C6, which are slightly different from the former clusters shown in Figure 2. The 3 and 7 dpa-enriched subsets are now clustered as C4 and C6, respectively. **b, c** Distribution of each sample's cells across the different core clusters. Cluster C2 expanded during regeneration, while clusters C0, C1, C3, C5, and C6 were reduced at 3 dpa before regaining relatively similar percentages as the uninjured control by 7 dpa. **d** Heatmap of top marker genes defining the clusters detected by both SC3 and Seurat. The order of the cells was determined by hierarchical clustering within each core cluster. *tcf21*, *aldh1a2*, and *hapln1a* expression are highlighted on top, but did not influence the cell ordering, which was driven by the genes within the heatmap. Normalized expression values are shown. **e** Biological process terms enriched for cluster marker genes in each cluster. The gene ratio is indicated by the dot size and the significance by the color of the dot ($P < 0.05$, one-sided hypergeometric test, see also Supplementary Data 3). The numbers of marker genes are listed in brackets under each cluster label. Notable GO terms include ECM organization (clusters C0 and C4), regeneration (C0, C1, C2, and C4), metabolic processes (C2 and C4), translation (C3, C4, and C5), and immune responses (C6). **f** Expression of selected marker genes on UMAPs (3 samples combined). **g** Pseudotime analysis results of the core epicardial clusters. Red arrows and letters highlight different trajectories. Clusters are labeled in the same number and color as in (a). **h** UMAPs showing expression patterns of genes correlated with the pseudotime trajectory.

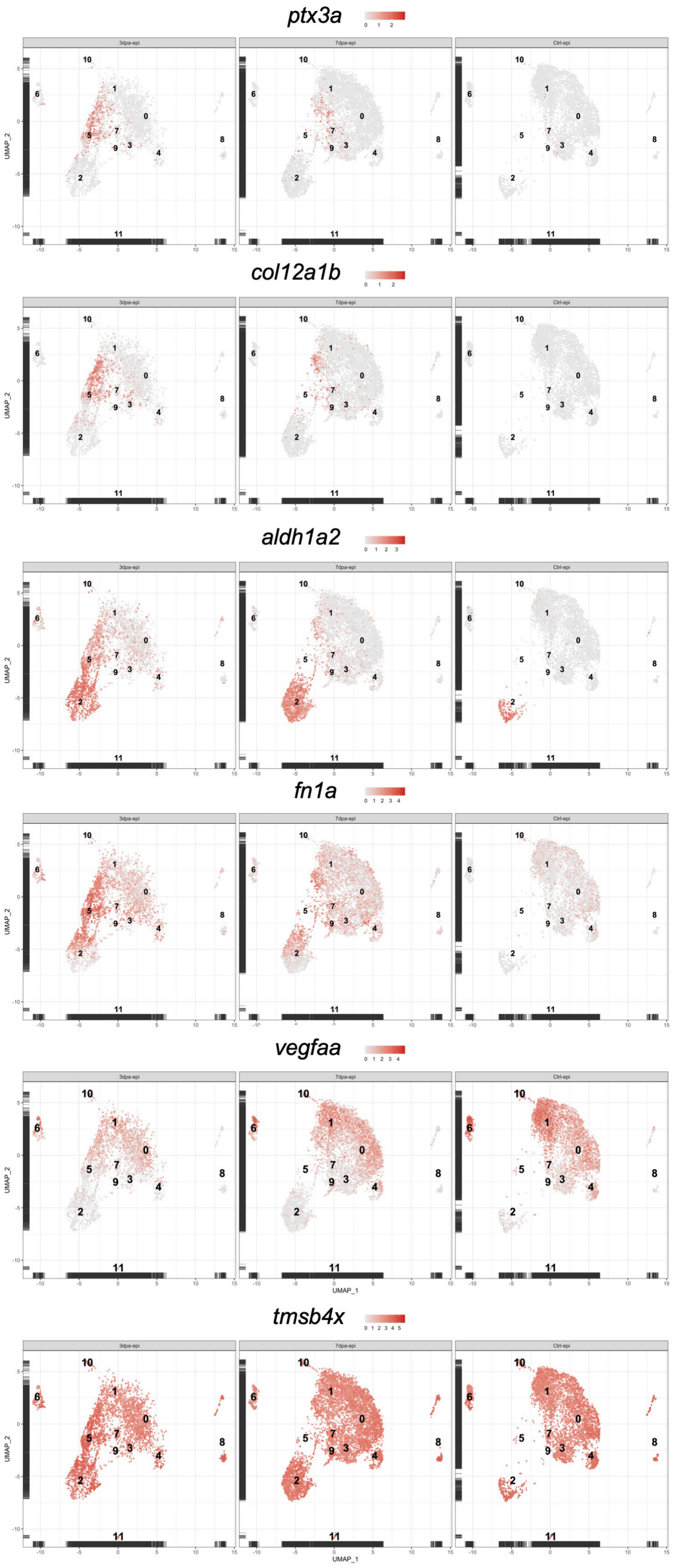
ptx3a
col12a1b
aldh1a2
fn1a
vegfaa
tmsb4x
3dpa-epi
7dpa-epi
Ctrl-epi
UMAP_1
UMAP_2

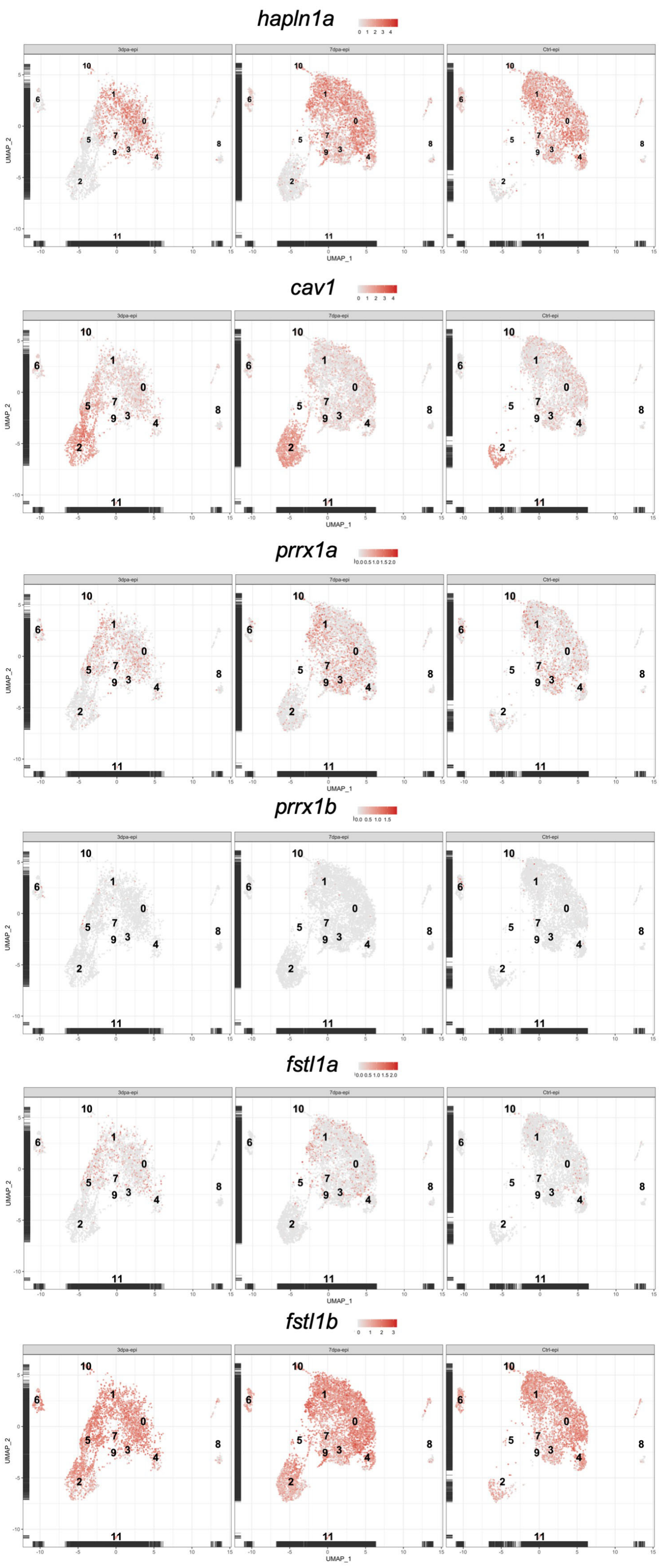

hapln1a
cav1
prrx1a
prrx1b
fstl1a
fstl1b
3dpa-epi
7dpa-epi
Ctrl-epi
UMAP_1
UMAP_2

Supplementary Figure 4. Expression patterns of selected genes across all clusters and samples.

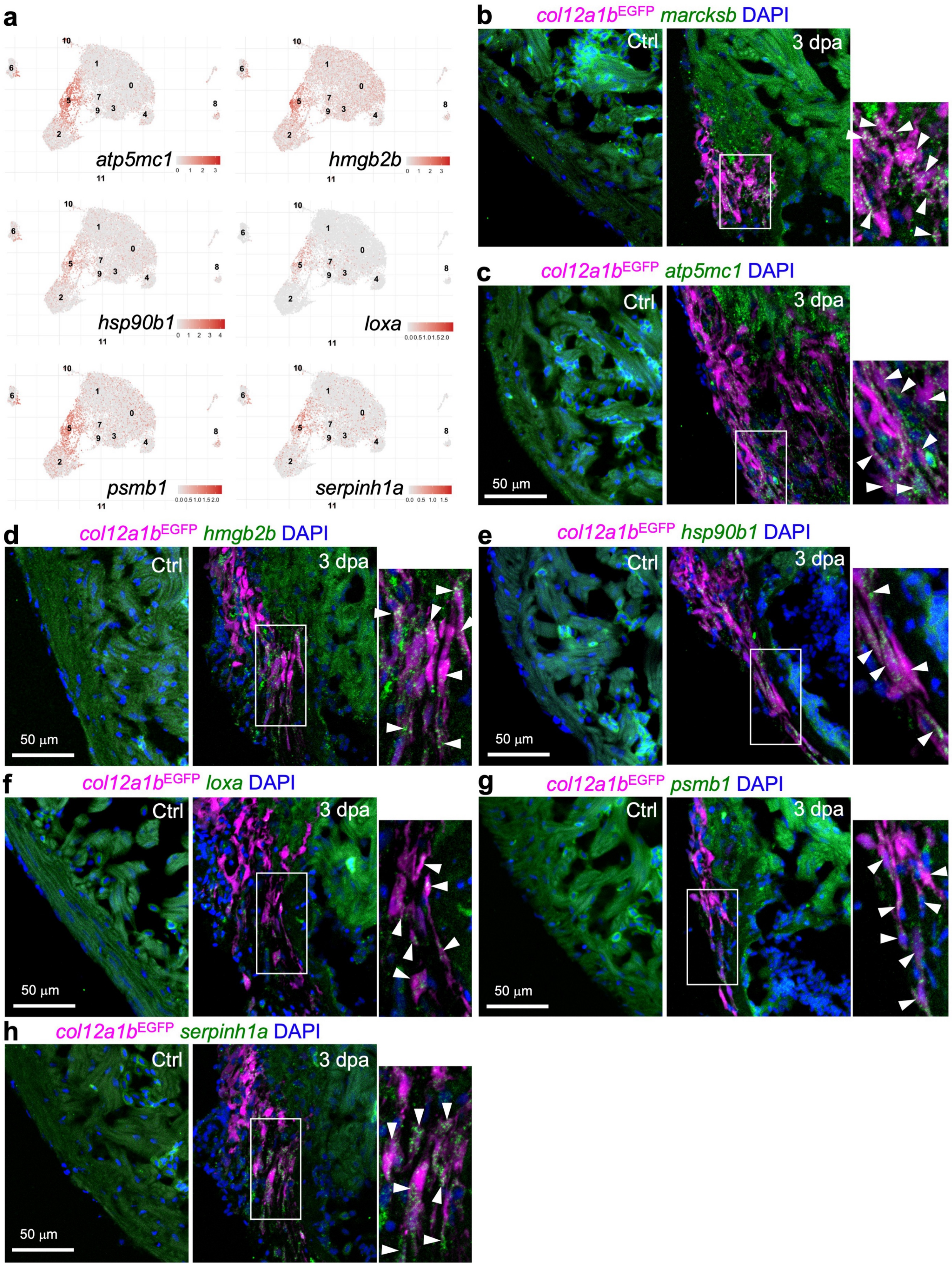

Supplementary Figure 5. Additional markers of aEPCs. a UMAPs showing gene

expressions. **b-h** Images of heart sections showing HCR staining results of selected aEPC markers in green, the *col12a1b*^{EGFP} reporter in magenta, and DAPI staining in blue. The injury sites of 3 dpa samples and the equivalent regions of the uninjured hearts (Ctrl) are shown. The framed regions are enlarged to show on the right with arrowheads indicating representative HCR staining signals in EGFP^{+} cells. Scale bar, 50 μm.

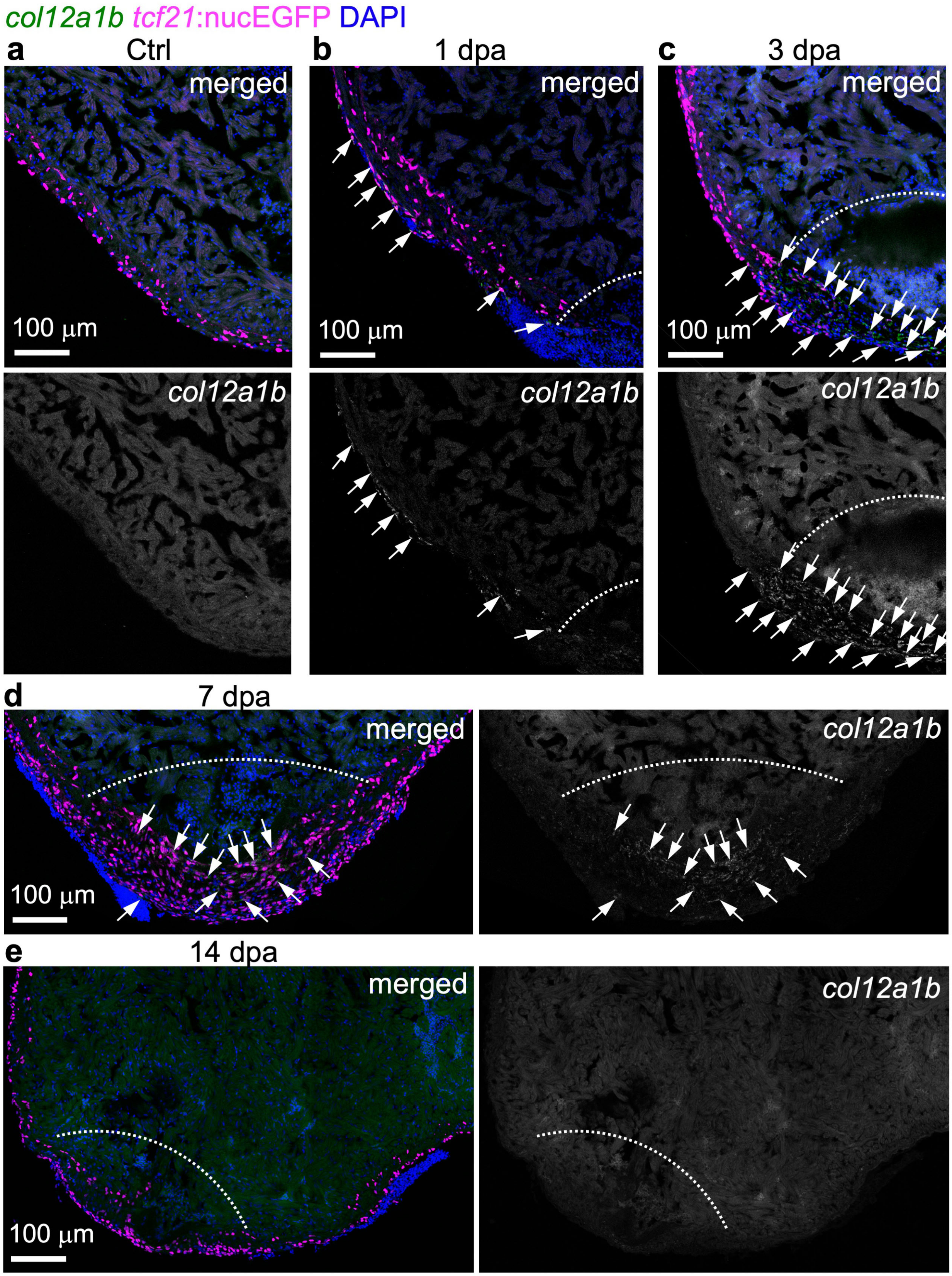

Supplementary Figure 6. *col12a1b* expression. HCR staining results of *col12a1b*

(green) on heart sections collected at 1 (**b**), 3 (**c**), 7 (**d**), and 14 dpa (**e**) together with the uninjured control (**a**). *tcf21*:nucEGFP (magenta) labels the epicardial cells. Nuclei were stained with DAPI (blue). Single-channel images show signals of *col12a1b*. White dashed lines indicate the injury sites. Arrows denote representative *col12a1b*$^{+}$EGFP^{+} cells. Scale bars, 100 μm.

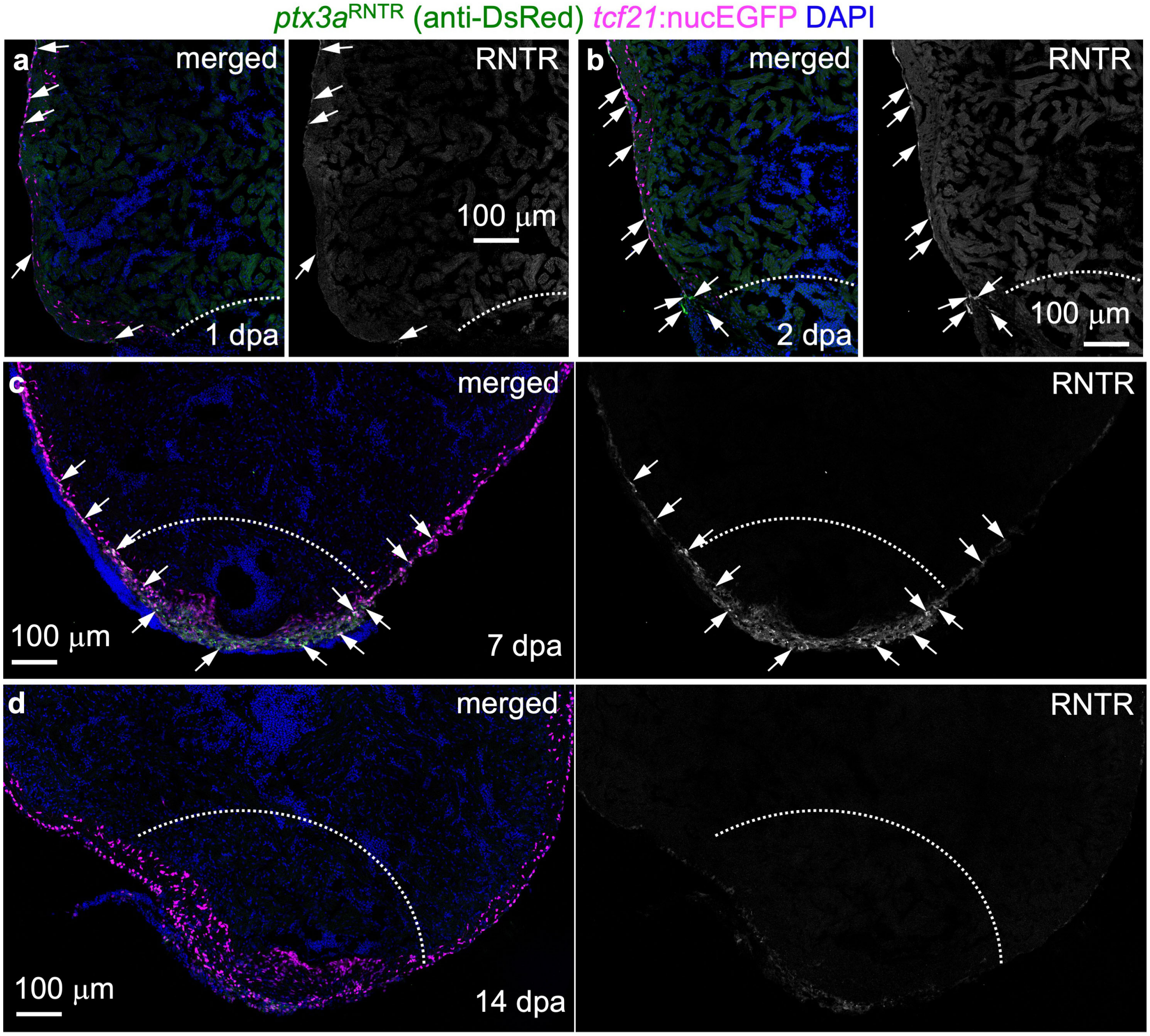

Supplementary Figure 7. Expression pattern of the *ptx3a^RNTR^* reporter line. Images of heart sections collected at 1 (**a**), 2 (**b**), 7 (**c**), and 14 dpa (**d**) showing expression of the *ptx3a*^{RNTR} reporter in green. *tcf21*:nucEGFP (magenta) labels the epicardial cells. Nuclei were stained with DAPI (blue). Single-channel images show signals of *ptx3a*^{RNTR} (with anti-DsRed antibody staining) on the right. White dashed lines indicate the injury sites. Arrows denote representative RNTR^{+}EGFP^{+} cells. Scale bars, 100 μm.

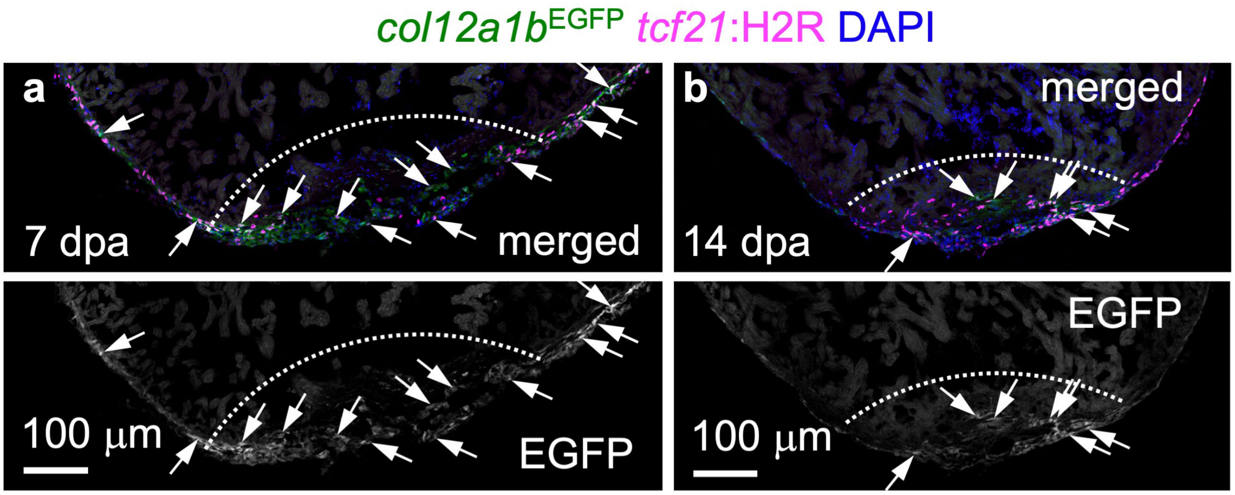

Supplementary Figure 8. Expression patterns of the *col12a1b*^{EGFP} reporter line. Images of heart sections collected at 7 (**a**) and 14 dpa (**b**) showing expression of the *col12a1b*^{EGFP} reporter in green. *tcf21*:H2R (magenta) labels the epicardial cells. Nuclei were stained with DAPI (blue). Single-channel images show signals of *col12a1b*^{EGFP}. White dashed lines indicate the injury sites. Arrows denote representative EGFP^{+}mCherry^{+} cells. Scale bars, 100 μm.

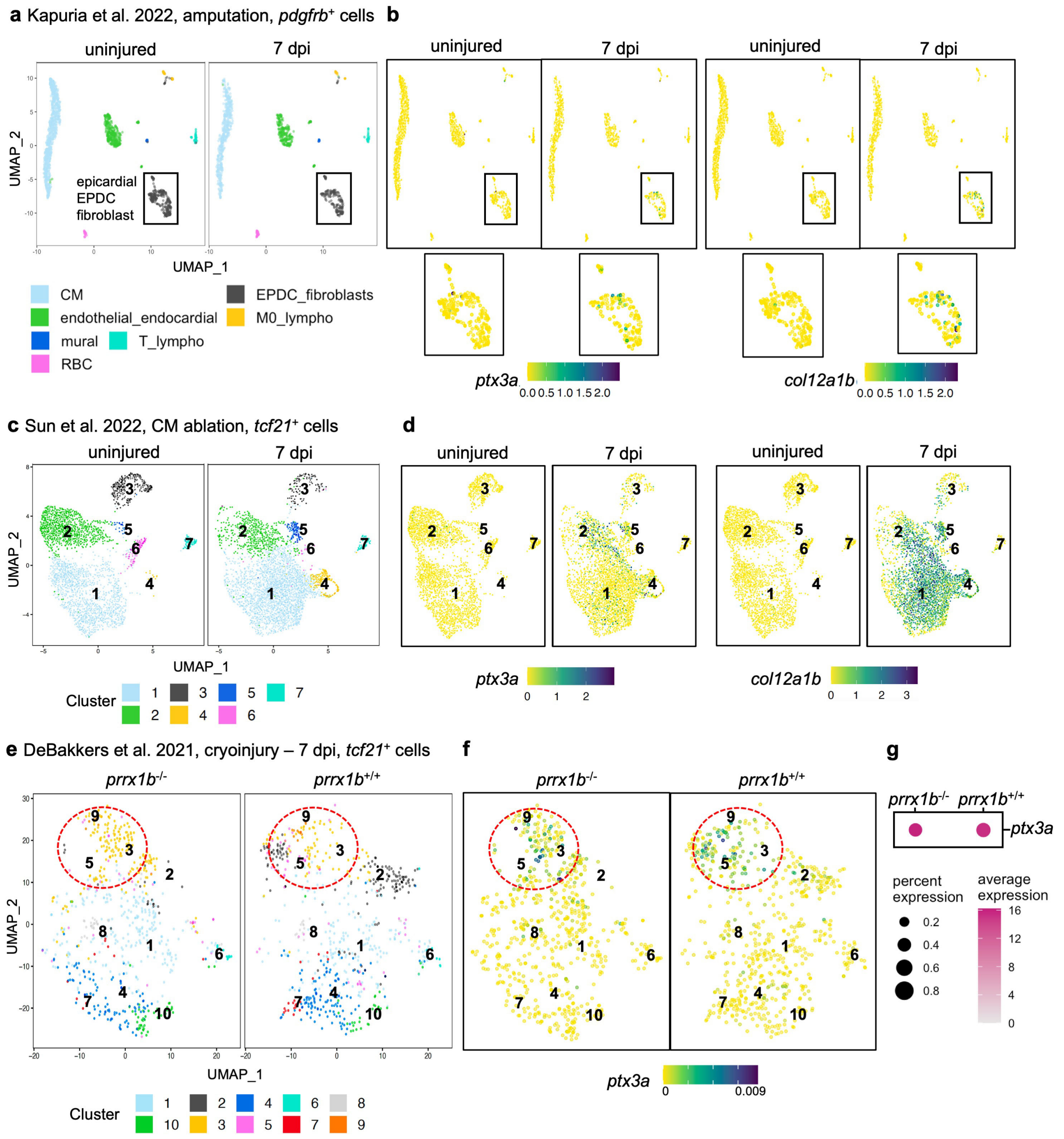

Supplementary Figure 9. Comparisons with other scRNA-seq datasets of zebrafish epicardium. a-b UMAPs of the Kapuria et al. 2022 dataset (amputation injury, isolated *pdgfrb*$^+$ cells) showing cell clusters (a) and gene expressions (b)**.** Expressions of *ptx3a* and *col12a1b* are induced in the epicardial/epicardial-derived cell (EPDC)/fibroblast cluster at 7 days post-injury (dpi). **c-d** UMAPs of the Sun et al. 2022 dataset (CM ablation injury, isolated *tcf21*$^+$ cells) showing cell clusters (c) and gene expressions (d)**.**

Expressions of *ptx3a* and *col12a1b* are induced by injury 7 dpi. **e-f** UMAPs of the DeBakkers et al. 2021 dataset (cryoinjury, isolated *tcf21*$^{+}$ cells) showing cell clusters (e) and gene expressions (f) in the wild type (*prrx1b*$^{+/+}$) and *prrx1b*$^{-/-}$ samples. *ptx3a* is expressed in clusters 2, 3, 5, and 9. **g** Dot plot showing percentages of cells expressing *ptx3a* (41% in the mutant versus 47% in the wild type) and the average expression levels.

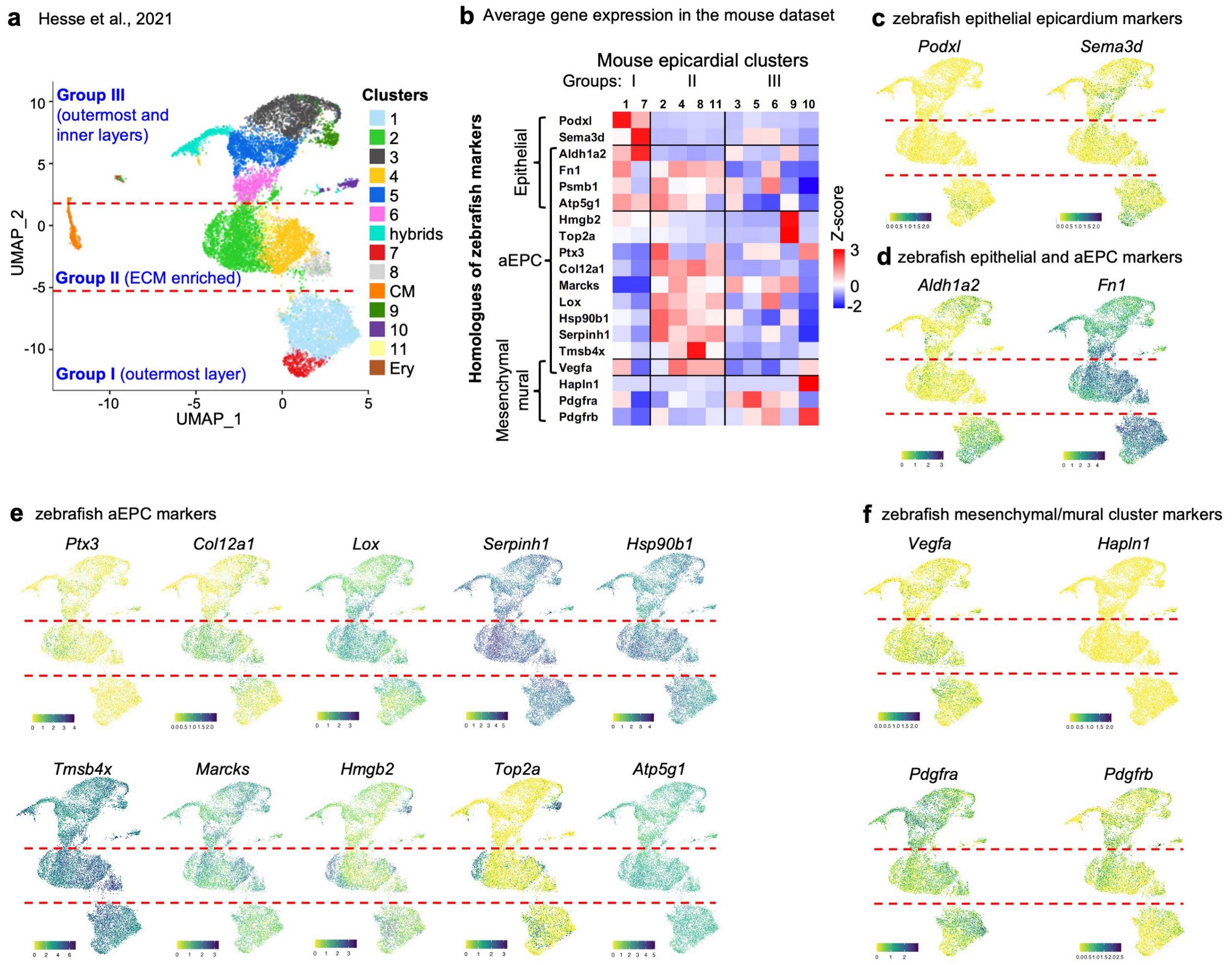

Supplementary Figure 10. Comparison with mouse epicardial stromal cells (EpiSC). **a** UMAPs of the Hesse et al. 2021 dataset showing cell clusters. Three groups of EpiSC were identified. Group I represents the outermost layer of the epicardium. Groups II and III cells reside in both the outermost and inner layers, and group II cells are enriched with expression ECM-related genes. **b** Heatmap showing average gene expressions of zebrafish epicardial cell markers in the mouse dataset. **c-f** UMAPs showing gene expressions of zebrafish epicardial cell markers in the mouse dataset.
