# Supplementary material for: Activation of a transient progenitor state in the epicardium is required for zebrafish heart regeneration
Source: Nat Commun. 2022 Dec 13;13:7704. doi: 10.1038/s41467-022-35433-9 (PMC9747719; doi:10.1038/s41467-022-35433-9)
Supplement: Supplementary file 3 — Description of Additional Supplementary Files [file 41467_2022_35433_MOESM3_ESM.pdf]

Description of Additional Supplementary Files

File Name: Supplementary Data 1
Description: List of marker genes for all clusters. For the markers identified by Seurat, FindAllMarkers function was used, which applys a two-sided Wilcoxon rank-sum test and p-values were adjusted with Bonferroni correction. pct.1 is the percentage of cells in the cluster where the gene is detected. pct.2 is the percentage of cells on average in all the other clusters where the gene is detected. For the markers identified by SC3, get_marker_genes function was used, which applys a two-sided Wilcoxon rank-sum test, and p-values were adjusted using the Hohm method. The area under the receiver operating characteristic (auroc) curve is used to quantify the accuracy of the prediction. The shared sheet shows markers identified by both methods.

File Name: Supplementary Data 2
Description: List of marker genes for the core epicardial clusters. The same methods were used as in Supplementary Data File 1.

File Name: Supplementary Data 3
Description: Enrichment annotation results of the marker genes for core epicardial clusters. GO terms were identified using the clusterProfiler enrichGO function, which uses a one-sided hypergeometric test. Adjusted p-values were calculated using the Benjamini-Hochberg procedure.
