# Supplementary material for: Activation of a transient progenitor state in the epicardium is required for zebrafish heart regeneration
Source: Nat Commun. 2022 Dec 13;13:7704. doi: 10.1038/s41467-022-35433-9 (PMC9747719; doi:10.1038/s41467-022-35433-9)
Supplement: Supplementary file 7 — Reporting Summary [file 41467_2022_35433_MOESM7_ESM.pdf]

nature portfolio

Corresponding author(s): Jingli Cao

Last updated by author(s): Nov 28, 2022

Reporting Summary

Nature Portfolio wishes to improve the reproducibility of the work that we publish. This form provides structure for consistency and transparency in reporting. For further information on Nature Portfolio policies, see our Editorial Policies and the Editorial Policy Checklist.

Statistics

For all statistical analyses, confirm that the following items are present in the figure legend, table legend, main text, or Methods section.

n/a	Confirmed	
☐	☒	The exact sample size (*n*) for each experimental group/condition, given as a discrete number and unit of measurement
☐	☒	A statement on whether measurements were taken from distinct samples or whether the same sample was measured repeatedly
☐	☒	The statistical test(s) used AND whether they are one- or two-sided<br>*Only common tests should be described solely by name; describe more complex techniques in the Methods section.*
☒	☐	A description of all covariates tested
☒	☐	A description of any assumptions or corrections, such as tests of normality and adjustment for multiple comparisons
☐	☒	A full description of the statistical parameters including central tendency (e.g. means) or other basic estimates (e.g. regression coefficient) AND variation (e.g. standard deviation) or associated estimates of uncertainty (e.g. confidence intervals)
☐	☒	For null hypothesis testing, the test statistic (e.g. *F*, *t*, *r*) with confidence intervals, effect sizes, degrees of freedom and *P* value noted<br>*Give P values as exact values whenever suitable.*
☒	☐	For Bayesian analysis, information on the choice of priors and Markov chain Monte Carlo settings
☒	☐	For hierarchical and complex designs, identification of the appropriate level for tests and full reporting of outcomes
☒	☐	Estimates of effect sizes (e.g. Cohen's *d*, Pearson's *r*), indicating how they were calculated

Our web collection on statistics for biologists contains articles on many of the points above.

Software and code

Policy information about availability of computer code

Data collection	Bright-field images of whole-mounted hearts were captured using a Zeiss Axiozoom V16 microscope (Zen 2.5 blue edition software). Fluorescent images of whole-mounted and sectioned heart tissues were imaged used a Zeiss 800 confocal microscope (Zen 2.6 blue edition software). AFOG staining images were captured on a Leica Dmi8 compound microscope (Leica Application Suite X software). Single cell RNA-sequencing were prepared on the 10x Chromium Single Cell instrument (10X Genomics) and sequenced on Illumina HiSeq4000.
Data analysis	Fluorescence images were exported from Zeiss Zen Blue (v2.5 or 2.6) software, processed in Adobe Photoshop 2022 and ImageJ (v2.0.0-rc-69/1.52p). Cell numbers and tissue thickness were quantified in Photoshop or ImageJ.<br><br>For scRNA-seq, CellRanger pipeline (v3.0.2) was used for raw data processing. Subsequent analyses were performed in R (v3.5.1) using functions provided in the R packages scater (v1.10.1) and scran (v1.16.0). Seurat v3.1.0 was used for data analyses and plotting, Monocle 3 (v0.1.3) was used to generate pseudotime trajectories. Marker genes were detected by Seurat's FindAllMarkers function and by SC3 (v1.10.1). Gene Ontology (GO) analysis was performed by using clusterProfiler (v3.10.1). All scripts as well as the code and cell labels used for generating the scRNA-seq based figures can be found at https://github.com/abcwcm/Cao_Epicardium.

For manuscripts utilizing custom algorithms or software that are central to the research but not yet described in published literature, software must be made available to editors and reviewers. We strongly encourage code deposition in a community repository (e.g. GitHub). See the Nature Portfolio guidelines for submitting code & software for further information.

Data

Policy information about availability of data

All manuscripts must include a data availability statement. This statement should provide the following information, where applicable:

- Accession codes, unique identifiers, or web links for publicly available datasets
- A description of any restrictions on data availability
- For clinical datasets or third party data, please ensure that the statement adheres to our policy

The scRNA-seq datasets have been deposited at NCBI's Gene Expression Omnibus under accession numbers GSE202836.

Human research participants

Policy information about studies involving human research participants and Sex and Gender in Research.

Reporting on sex and gender	N/A
Population characteristics	N/A
Recruitment	N/A
Ethics oversight	N/A

Note that full information on the approval of the study protocol must also be provided in the manuscript.

Field-specific reporting

Please select the one below that is the best fit for your research. If you are not sure, read the appropriate sections before making your selection.

☒ Life sciences ☐ Behavioural & social sciences ☐ Ecological, evolutionary & environmental sciences

For a reference copy of the document with all sections, see nature.com/documents/nr-reporting-summary-flat.pdf

Life sciences study design

All studies must disclose on these points even when the disclosure is negative.

Sample size	Sample sizes were chosen based on previous publications (such as PMIDs: 35179181, 25938716, and 35652354) and experiment types and are indicated in each figure legends.<br>For all fluorescent images showing representative results, at least 6 zebrafish were analyzed with consistent results.<br>For the quantification of modRNA injection result (Fig. 1f), 6 or 13 samples were analyzed in each group.<br>For the evaluation of wound healing after heart injury (Fig. 7b and 9d), 6-18 samples were analyzed for each group with consistent patterns present in most samples (as noted in the Figure panels).<br>For the quantification of epicardial cell repopulation of the wound (Fig. 7d), 7-8 hearts were used for each group.<br>For the cardiomyocyte proliferation analysis (Fig. 7f), 14-16 samples were counted for each group.<br>For the evaluation of histological recovery (Fig. 7h), 13 hearts were assessed for each group.<br>For the quantification of nrg1 signal in the wound (Fig. 8b) and mesenchymal cells in the wound (Fig. 8d), 8-13 samples were counted for each group.<br>For the quantifications of TGFb-inhibition phenotypes (9f-h, and j), 6 or 7 hearts were used for each group.<br>These samples sizes ensure statistic power ($P < 0.05$) that matches biological observations.
Data exclusions	No animal or sample was excluded from the analysis unless the animal died during the procedure.
Replication	All experiments were performed successfully with similar results from at least 2 biological replicates.
Randomization	Clutchmates, or hearts collected from clutchmates, were randomized into different groups for each treatment.
Blinding	The investigators were blinded to group allocation during data collection but not during data analysis because the quantitative results were not influenced by investigator's bias.

Reporting for specific materials, systems and methods

We require information from authors about some types of materials, experimental systems and methods used in many studies. Here, indicate whether each material, system or method listed is relevant to your study. If you are not sure if a list item applies to your research, read the appropriate section before selecting a response.

Materials & experimental systems

n/a	Involved in the study
	☒ Antibodies
☒	☐ Eukaryotic cell lines
☒	☐ Palaeontology and archaeology
	☒ Animals and other organisms
☒	☐ Clinical data
☒	☐ Dual use research of concern

Methods

n/a	Involved in the study
☒	☐ ChIP-seq
☒	☐ Flow cytometry
☒	☐ MRI-based neuroimaging

Antibodies

Antibodies used

Primary antibodies: rabbit anti-Mef2 (Boster Bio, DZ01398-1; 1:200), mouse anti-PCNA (Sigma, P8825, RRID:AB_477413; 1:350), rabbit anti-Aldh1a2 (GeneTex, GTX124302, RRID:AB_11177627; 1:350), rabbit anti-DsRed (Takara, 632496, RRID:AB_10013483; 1:200), mouse anti-Tnnt (ThermoFisher, MS-295-PABX, RRID:AB_61810; 1:100)
Secondary antibodies (used at 1:200): Alexa Fluor 488 goat anti-rabbit (ThermoFisher, A11034, RRID:AB_2576217) and goat anti-mouse (ThermoFisher, A11029, RRID:AB_2534088) , Alexa Fluor 546 goat anti-rabbit (ThermoFisher, A11035, RRID:AB_2534093) and goat anti-mouse (ThermoFisher, A11030, RRID:AB_2534089) , Alexa Fluor 633 goat anti-mouse (ThermoFisher, A21052, RRID:AB_2535719).

Validation

All primary antibodies are commercially available and are validated by suppliers as follows. All antibodies used in this study have been widely used with multiple publications.
rabbit anti-Mef2: https://www.bosterbio.com/polyclonal-anti-mef2-antibody-dz01398-1-boster.html
mouse anti-PCNA: https://www.sigmaaldrich.com/US/en/product/sigma/p8825
rabbit anti-Aldh1a2: https://www.genetex.com/Product/Detail/Aldh1a2-antibody/GTX124302
rabbit anti-DsRed: https://www.takarabio.com/products/antibodies-and-elisa/fluorescent-protein-antibodies/red-fluorescent-protein-antibodies
mouse anti-Tnnt: https://tools.thermofisher.com/content/sfs/brochures/D11737~.pdf

Animals and other research organisms

Policy information about studies involving animals; ARRIVE guidelines recommended for reporting animal research, and Sex and Gender in Research

Laboratory animals	Zebrafish of the Ekkwill (EK) and Ekkwill/AB strains between 3 and 12-month old were used.
Wild animals	No wild animal used
Reporting on sex	Animals of both sexes were used for experiments
Field-collected samples	No field-collected samples
Ethics oversight	Animal procedures were approved by the Institutional Animal Care and Use Committee (IACUC) at Weill Cornell Medical College.

Note that full information on the approval of the study protocol must also be provided in the manuscript.
